# Aortic carboxypeptidase-like protein regulates vascular adventitial progenitor and fibroblast differentiation through myocardin related transcription factor A

**DOI:** 10.1038/s41598-021-82941-7

**Published:** 2021-02-17

**Authors:** Dahai Wang, Nabil Rabhi, Shaw-Fang Yet, Stephen R. Farmer, Matthew D. Layne

**Affiliations:** 1 Department of Biochemistry, Boston University School of Medicine, 72 E. Concord St, Boston, MA 02118 USA; 2 Institute of Cellular and System Medicine, National Health Research Institutes, Zhunan, 35053 Taiwan; 3 Department of Hematology, Boston Children’s Hospital, Boston, MA USA

**Keywords:** Mechanisms of disease, Transcription factors, Cell biology, Cell signalling, Extracellular signalling molecules, Cardiovascular biology

## Abstract

The vascular adventitia contains numerous cell types including fibroblasts, adipocytes, inflammatory cells, and progenitors embedded within a complex extracellular matrix (ECM) network. In response to vascular injury, adventitial progenitors and fibroblasts become activated and exhibit increased proliferative capacity and differentiate into contractile cells that remodel the ECM. These processes can lead to vascular fibrosis and disease progression. Our previous work established that the ECM protein aortic carboxypeptidase-like protein (ACLP) promotes fibrotic remodeling in the lung and is activated by vascular injury. It is currently unknown what controls vascular adventitial cell differentiation and if ACLP has a role in this process. Using purified mouse aortic adventitia Sca1+ progenitors, ACLP repressed stem cell markers (CD34, KLF4) and upregulated smooth muscle actin (SMA) and collagen I expression. ACLP enhanced myocardin-related transcription factor A (MRTFA) activity in adventitial cells by promoting MRTFA nuclear translocation. Sca1 cells from MRTFA-null mice exhibited reduced SMA and collagen expression induced by ACLP, indicating Sca1 cell differentiation is regulated in part by the ACLP-MRTFA axis. We determined that ACLP induced vessel contraction and increased adventitial collagen in an explant model. Collectively these studies identified ACLP as a mediator of adventitial cellular differentiation, which may result in pathological vessel remodeling.

## Introduction

The adventitia, the outermost layer of muscular and elastic arteries, is bounded from the medial smooth muscle cell (SMC) layer by the external elastic lamina^1–3^. Originally considered a simple supportive structure, the adventitia is emerging as a key regulator of vascular diseases including atherosclerosis^1,4,5^ and pulmonary vascular hypertension^2,6^. The adventitia is an assembly of several cell types including fibroblasts, stem/progenitor cells, adipocytes, neurons, and inflammatory cells and in the normal vessel, these cells are organized within a connective tissue rich in fibrillar collagens types I and III^7^. In response to injury, adventitial fibroblasts and progenitor cells are activated. The activation of proliferation, migration, and differentiation programs eventually leads to adventitial fibrosis that may restrict beneficial vessel expansion^8–10^. Due in part to the complexity of the adventitia, the mechanisms controlling adventitial cell differentiation and remodeling have yet to be elucidated.

There are numerous similarities between fibroblast to myofibroblast differentiation and progenitor to vascular smooth muscle differentiation pathways with the notable expression of the contractile, cytoskeletal protein α-smooth muscle actin (SMA)^11^. In fibrotic diseases, differentiated myofibroblasts secrete collagens, including type I, and subsequently remodel it through their contractile functions. Myofibroblasts participate in vascular repair processes but also result in adventitial extracellular matrix (ECM) accumulation^1,2^. These same processes can also result in inward arterial remodeling, which potentiates tissue ischemia, myocardial infarction, and stroke^12^. Myofibroblast differentiation is regulated by numerous growth factors including transforming growth factor β (TGFβ) and ECM molecules including collagen and fibronectin^13^. The ECM influences myofibroblast differentiation through both biochemical and mechanical signaling^11,13,14^. Importantly, the transcriptional regulator, myocardin-related transcription factor A (MRTFA/Mkl1) acts as a central regulator of myofibroblast differentiation and fibrosis^15,16^. In response to extracellular stimuli, changes in cytoskeletal reorganization and actin polymerization can induce MRTFA nuclear translocation and transcriptional activity including the regulation of collagen^17,18^.

While adventitial fibroblasts are clearly important in vascular remodeling, adventitial stem cell antigen-1 (Sca1) positive progenitor cells adopt both vascular and nonvascular fates^19–22^. Adventitial Sca1 cells in adult mice with hypertension can give rise to collagen-producing cells and contribute to vascular fibrosis^23^ and Sca1 cells in the coronary adventitia are responsible for perivascular fibrosis^20^. In an atherosclerosis model in mice, progenitor cells in the adventitia differentiate into smooth muscle-like cells and contribute to vein graft atherosclerosis^19^. Importantly, adventitial Sca1 progenitors are maintained by sonic hedgehog signaling^24^ and when activated contribute to both vascular fibrosis and cardiovascular disease. However, the complex pathways controlling their differentiation are only partially understood.

Our previous studies identified a secreted protein, aortic carboxypeptidase-like protein (ACLP/*AEBP1*), which contains an evolutionarily conserved collagen binding discoidin domain and a catalytically inactive carboxypeptidase domain^25–27^. ACLP is highly expressed and secreted by fibroblasts and injured SMCs^25–28^. Several studies identified correlations of ACLP expression with cardiovascular disease processes. For example, ACLP was discovered in the ECM in a porcine model of ischemia/reperfusion cardiac remodeling^29^, in human abdominal aortic aneurysms^30^, and in the plasma following cardiac injury^31^. Our work has shown ACLP expression increases with SMC development^26,32^ and at sites of tissue injury^25,26,28^. In addition, mutations in the *AEBP1* gene that encodes ACLP result in Ehlers Danlos Syndrome with vascular complications^33,34^. Taken together, there is strong evidence correlating ACLP with vascular disease processes. However, little is currently known about ACLP’s role in controlling adventitial cell biology. In this study, we show that ACLP promotes adventitial progenitor and fibroblast differentiation. We identified a novel pathway where ACLP-induced Sca1 cell differentiation is controlled in part by enhancing MRTFA nuclear localization and activity.

## Results

### ACLP promotes Sca1 cell differentiation into myofibroblast-like cells

**Figure 1:**
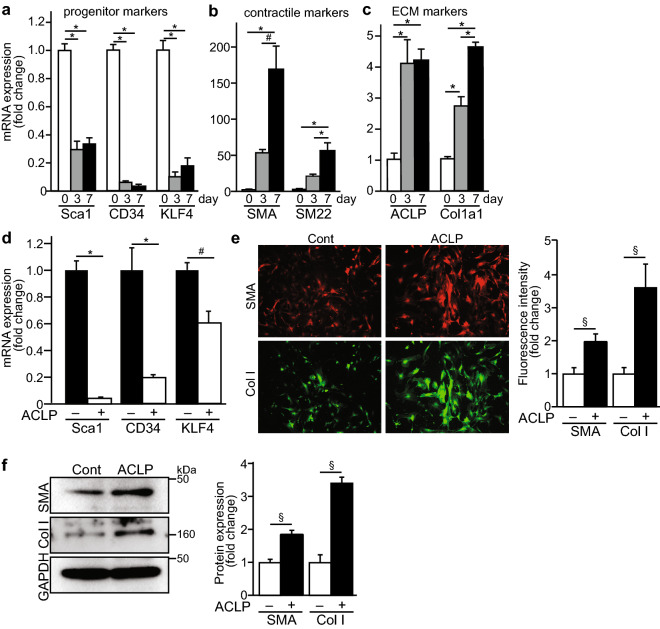
ACLP promotes adventitial Sca1 cell differentiation into myofibroblast-like cells. Sca1 cells were isolated from mouse aorta adventitia and cultured on tissue culture plastic and RNA was isolated at the given times and analyzed by qRT-PCR (**a**) progenitor marker genes (**b**) smooth muscle/contractile marker genes; (**c**) ECM marker genes. Results are presented as the mean ± SEM for 3 independent experiments performed in triplicate. Statistical significance was calculated using ANOVA, followed by Tukey’s multiple comparison test. *p < 0.001, ^#^p < 0.005 and ^§^p < 0.05. Sca1 cells were isolated from the aortic adventitia of SMA-mCherry and col3.6GFPtpz transgenic male mice and cultured for 7 days. (**d**) Progenitor marker genes were examined by RT-PCR. Results are presented as the mean ± SEM for 3 independent experiments performed in triplicate. Data were analyzed by ANOVA, followed by Tukey’s multiple comparison test. *p < 0.001, ^#^*p* < 0.005. (**e**) Images of SMA-mCherry and col3.6GFPtpz reporter expression were taken by fluorescence microscopy. Representative images are shown from three independent experiments. Fluorescence intensity of reporter expression was analyzed by ImageJ and normalized by control. Data were analyzed by the Student’s t test. ^§^*p* < 0.05. (**f**) Protein expression was examined by Western blot (left) and quantification of results from 3 independent experiments (right). Statistical significance was calculated using the Student’s t test (two tailed). ^§^*p* < 0.05.

It has been established that resident Sca1 positive progenitor cells in adventitia have a potential to switch their phenotype and differentiate into collagen-producing cells or smooth muscle-like cells^19,23^. In the adventitial niche, Sca1+ cells are heterogeneous and contribute to vascular disease and remodeling^24,35,36^. It is also recognized that the ECM is an essential regulator of the stem cell niche and interacts with progenitor cells to determine their differentiation capacity^37,38^. ACLP is a secreted ECM protein and we have previously demonstrated that ACLP promotes lung fibroblast differentiation and enhances adipose tissue stromal progenitor differentiation^28,39,40^. To understand the mechanisms controlling the differentiation of adventitial Sca1 progenitors with respect to contractile and ECM markers, we purified mouse vascular adventitial Sca1+ cells using microbeads. These Sca1+ cells were characterized by high expression of progenitor markers including Sca1, CD34 and transcription factor krüppel-like factor 4 (KLF4) (Fig. 1a). In culture, these cells progressively lose progenitor marker expression (Sca1, KLF4, CD34) with significant reductions by day 3 (Fig. 1a). Concomitant with the loss of stem cell markers, these cells differentiate into myofibroblast or smooth muscle-like cells characterized by significant increases in smooth muscle/myofibroblast marker gene expression including SMA and SM22α by day 7 (Fig. 1b). Importantly, as these cells differentiate, they also significantly increase their expression of ACLP and collagen I (Fig. 1c).

Because ACLP was upregulated during progenitor differentiation and we also know that it regulates both SMA and collagen expression in fibrotic cells, we next examined whether exogenous ACLP could enhance adventitial progenitor differentiation. To do this we studied adventitial Sca1 cells isolated from transgenic mice harboring the SMA-mCherry and collagen1-GFP-Tpz transgenes to monitor changes in gene expression. After treatment with ACLP for 7 days, we isolated RNA and expression was analyzed by qRT-PCR. We found that compared with controls, ACLP further decreased Sca1, CD34, and KLF4 gene expression (Fig. 1d). Consistent with mRNA level changes, ACLP treatment increased SMA and collagen I reporter expression using live cell imaging and quantified using ImageJ (Fig. 1e). Protein isolates from these cells revealed an increase in both collagen I and SMA by Western blot analysis (Fig. 1f). Taken together these data indicate that ACLP down regulates progenitor marker expression and promotes differentiation into myofibroblasts.

### ACLP promoting Sca1 cell differentiation is mediated by MRTFA pathway

**Figure 2:**
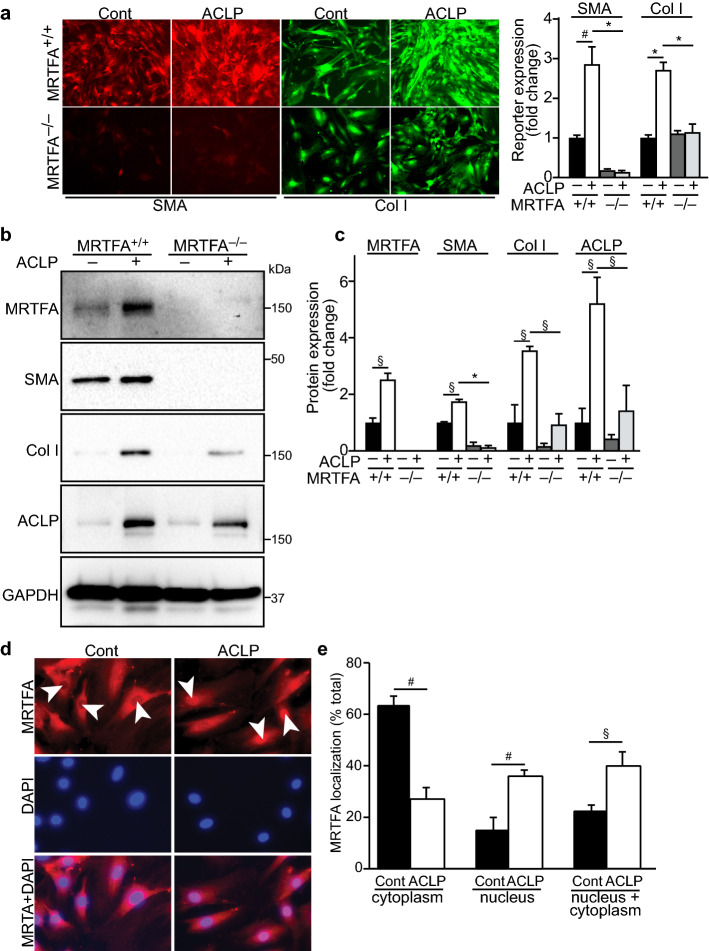
ACLP promotes Sca1 cell differentiation via the MRTFA pathway by promoting MRTFA nuclear translocation. Sca1 cells were isolated from MRTFA^+/+^ and MRTFA^−/−^ mouse aorta adventitia and cultured in the presence or absence of ACLP (30 nM) for 7 days. (**a**) SMA-mCherry and col3.6GFPtpz reporter activity was monitored by fluorescence microscopy. Representative images are shown from three independent experiments. Fluorescence intensity of reporter expression was analyzed by ImageJ and normalized by control. Data were analyzed by ANOVA, followed by Tukey’s multiple comparison test. *p < 0.001, ^#^*p* < 0.005. (**b**) Adventitial fibroblast protein expression was examined by Western blot. (**c**) Quantification of Western results from three independent experiments. Statistical significance was calculated using ANOVA, followed by Tukey’s multiple comparison test. *p < 0.001, ^#^*p* < 0.005 and ^§^*p* < 0.05. Adventitial fibroblasts were serum-starved overnight and treated with ACLP (30 nM) for 2 h, MRTFA nuclear translocation was examined by immunofluorescence staining (**d**) and quantified as shown in (**e**). Data shown represent the mean ± SEM from 3 independent experiments. Statistical significance was calculated using the Student’s t test (two tailed). ^§^*p* < 0.05, ^#^*p* < 0.005.

Our previous work and that of others supports the concept that MRTFA regulates type I collagen and myofibroblast gene expression through transcriptional complexes with both serum response factor (SRF) and Sp1^17,18^. The MRTFA pathway is also required for TGFβ-induced fibroblast differentiation^15,41,42^. To explore whether adventitial progenitor differentiation required MRTFA activity and more specifically if ACLP stimulated this pathway, we isolated adventitial Sca1 progenitors from MRTFA^+/+^ and MRTFA^−/−^ mouse aorta and cultured in the presence or absence of ACLP. We found that ACLP promoted Sca1 differentiation into myofibroblasts by increasing myofibroblast-associated protein SMA and collagen I expression. Importantly, using live cell imaging of the SMA and collagen I reporters, both were blunted in cells differentiated from MRTFA^−/−^ Sca1 progenitors compared with controls (Fig. 2a). Consistent with the collagen and SMA reporter fluorescence, Western blotting showed that compared with MRTFA^+/+^ fibroblasts, MRTFA deficient fibroblasts exhibited reduced ACLP stimulated SMA, collagen I, and ACLP expression (Fig. 2b,c). Notably, ACLP stimulated its own expression in these cells. Taken together these findings indicate that ACLP-induced Sca1 cell differentiation is mediated in part through the MRTFA pathway.

MRTFA activity is regulated through its interaction with G-actin in the cytoplasm via its RPEL domain^43^. In response to several stimuli including actin polymerization, MRTFA is released and rapidly translocates to the nucleus to enhance expression of its target genes^15^. We next asked whether ACLP regulates MRTFA activity by measuring nuclear translocation of MRTFA. Adventitial fibroblasts were treated with ACLP for 2 h and MRTFA nuclear translocation was examined by immunofluorescence staining. In the untreated control fibroblasts, MRTFA exhibited predominantly cytoplasmic location (Fig. 2d). Compared with controls, ACLP treatment increased MRTFA nuclear accumulation and more ACLP treated cells exhibited MRTFA expression in both the nucleus and cytoplasm (Fig. 2e).

### ACLP stimulates fibroblast-to-myofibroblast differentiation

**Figure 3:**
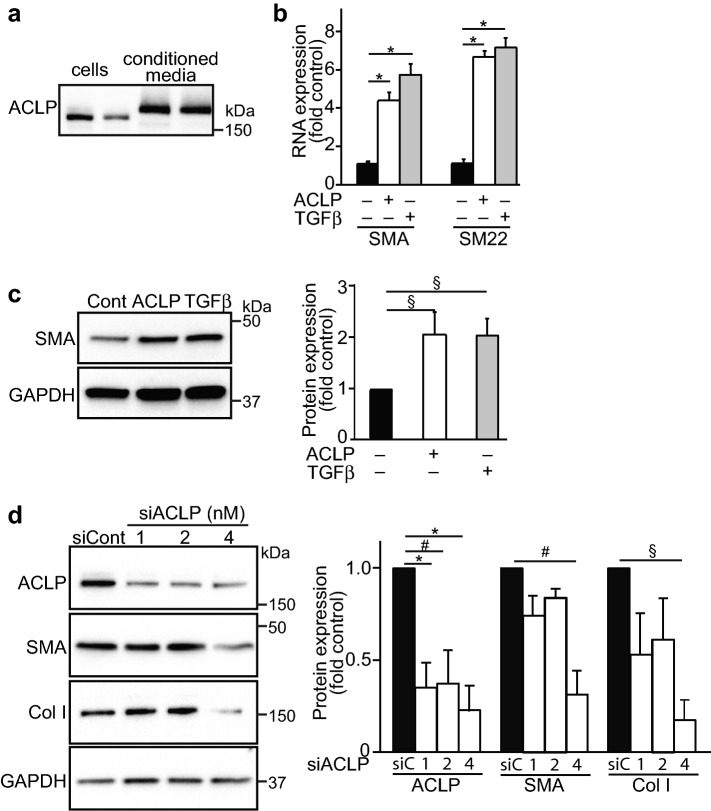
ACLP regulates myofibroblast marker gene expression. Fibroblasts were isolated from mouse adventitia and cultured in 0.5% FBS/DMEM. Two days later, cells and medium were collected and ACLP expression examined by Western blot (**a**). Mouse adventitial fibroblasts were treated with ACLP (30 nM) or TGFβ (1 nM) for 24 h. SMA (Acta2) and SM22 mRNA were examined by RT-PCR. Results are presented as the mean ± SEM for 3 independent experiments performed in triplicate (**b**). SMA protein was examined by Western Blot (**c**) and data are presented as the mean ± SEM for 3 independent experiments and analyzed by one way ANOVA with post hoc Tukey’s test. Mouse adventitial fibroblasts were transfected with different doses of siACLP or siControl. (**d**) Protein was extracted 48 h later and subjected to Western blot. Protein expression was quantified by densitometry normalized to GAPDH expression and relatively compared to untreated control cells (3 independent experiments). Statistical significance was calculated using ANOVA, followed by Tukey’s multiple comparison test *p < 0.001, ^#^*p* < 0.005, ^§^*p* < 0.05.

In addition to specific progenitors, adventitial fibroblasts also contribute to vascular remodeling^1,2,44^. Furthermore, the differentiated Sca1 cells take on properties similar to activated adventitial fibroblast^24^. We next studied these adventitial fibroblasts in culture and found that they express and secrete high levels of ACLP (Fig. 3a). The upper band in these blots is the glycosylated, secreted form of ACLP^28^. Similar to the experiments with Sca1 cells, mouse adventitial fibroblasts treated with ACLP or TGFβ (a positive control and known mediator of fibroblast-to-myofibroblast differentiation) significantly increased SMA and SM22 mRNA expression (Fig. 3b). Consistent with these results, ACLP also enhanced SMA protein expression (Fig. 3c). Using a siRNA loss of function approach, we found that knocking down ACLP significantly decreased SMA and collagen I protein expression (Fig. 3d). These data indicate that adventitial fibroblasts synthesize ACLP and ACLP promotes fibroblast-to-myofibroblast transition in these cells by enhancing SMA and collagen I expression.

### ACLP potentiates collagen gel contraction by fibroblasts

**Figure 4:**
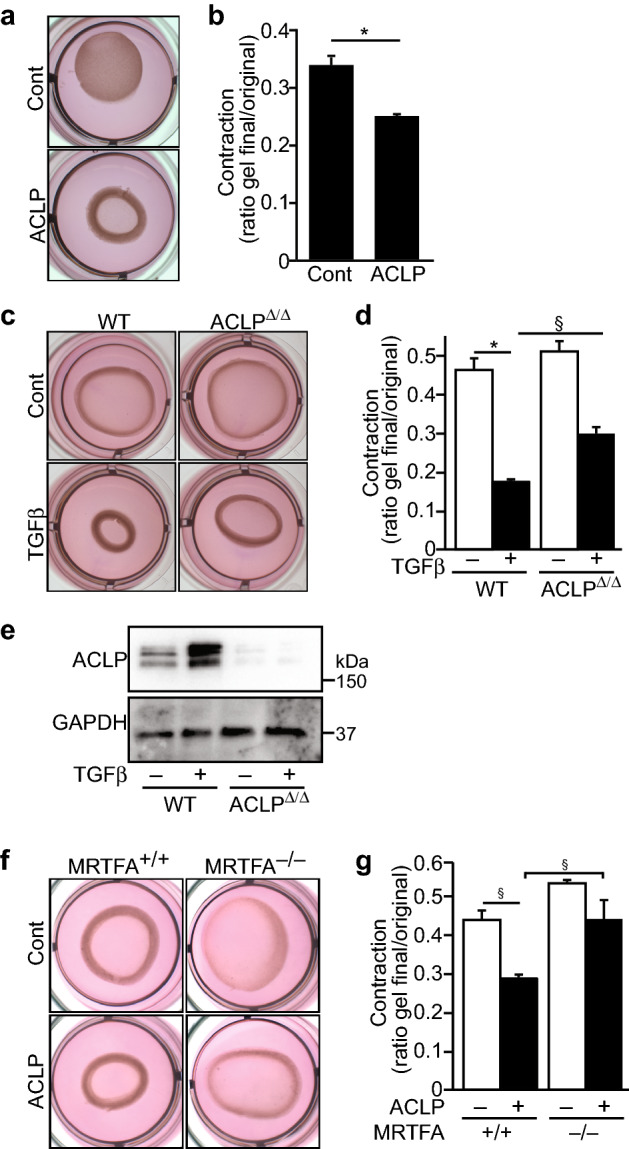
ACLP-induced gel contraction in fibroblasts is blunted by loss of MRTFA. (**a**) Mouse adventitial fibroblasts were plated into collagen gels in the presence or absence of ACLP (30 nM), followed by overnight incubation. The area of contracted collagen lattices was calculated as a percentage of the surface of the dish (**b**). Statistical significance was calculated using the Student’s t test (two tailed) (n = 3). Mouse adventitial fibroblasts were isolated from ACLP^flx/flx^·Cre+ mice. After ACLP deletion by tamoxifen treatment, cells were plated into a collagen gel in the presence or absence of TGFβ (1 nM) for collagen gel contraction assays (**c**). The area of contracted collagen lattices was calculated as a percentage of the surface of the dish (**d**). ACLP protein expression was analyzed by Western Blot (**e**). Results shown represent the mean ± SEM (n = 3). Statistical significance was calculated using ANOVA, followed by Tukey’s multiple comparison test *p < 0.001, ^§^*p* < 0.05. (**f**) Adventitial fibroblasts from MRTFA^+/+^ and MRTFA^−/−^ male mice were plated into collagen gels in the presence or absence of ACLP (30 nM), followed by overnight incubation. The area of contracted collagen lattices was calculated as a percentage of the surface of the dish (**g**). Data shown represent the mean ± SEM (n = 3). Statistical significance was calculated using one way ANOVA with post hoc Tukey’s test. ^§^*p* < 0.05.

In addition to changes in cellular composition, in vascular disease adventitial fibrosis is characterized by the accumulation of collagen, which is then remodeled through cellular contractile activity^1–3^. We hypothesized that ACLP in the collagen-rich ECM surrounding adventitial fibroblasts promotes ECM contraction induced by fibroblasts. Mouse adventitial fibroblasts were plated into collagen gels in the presence or absence of ACLP, and then incubated overnight. The area of contracted collagen lattices was calculated as a percentage of the initial size. ACLP induced gel contraction by reducing gel size from 33 to 24% of their original size compared to controls, indicating that ACLP promotes fibroblast-induced gel contraction (Fig. 4a,b). We recently generated mice with a floxed ACLP allele (ACLP^flx/flx^) and crossed these mice with a tamoxifen controlled Cre mice^45^. After ACLP deletion by tamoxifen treatment in vitro, cells were plated into collagen gels in the presence or absence of TGFβ to stimulate contraction. As expected, TGFβ induced collagen gel contraction in ACLP^+/+^ fibroblasts. This effect was blunted in the ACLP^Δ/Δ^ adventitial fibroblasts (Fig. 4c,d). Western blotting revealed that TGFβ increased ACLP levels in ACLP^+/+^ cells and as expected ACLP^Δ/Δ^ fibroblasts lacked ACLP expression (Fig. 4e). Taken together, these data indicate that ACLP potentiates collagen gel contraction by fibroblasts and ACLP-containing adventitial matrix participates in regulating ECM contraction. To assess the requirement of MRTFA in ACLP mediated collagen gel contraction, adventitial fibroblasts were isolated from MRTFA^+/+^ and MRTFA^−/−^ mice and were plated in collagen gels in the presence or absence of ACLP. Similar to previous results (Fig. 4a) we observed a significant enhancement of gel contraction in the MRTFA^+/+^ cells (Fig. 4f,g). However, both untreated and ACLP-induced gel contraction was blunted in the MRTFA^−/−^ cells compared to MRTFA^+/+^ cells (Fig. 4f,g). In addition, contraction was not significantly different between control and ACLP treated MRTFA^−/−^ cells (Fig. 4f,g) further supporting the concept MRTFA is an important mediator of collagen contractility.

### ACLP induces adventitial fibrosis and aorta contraction

**Figure 5:**
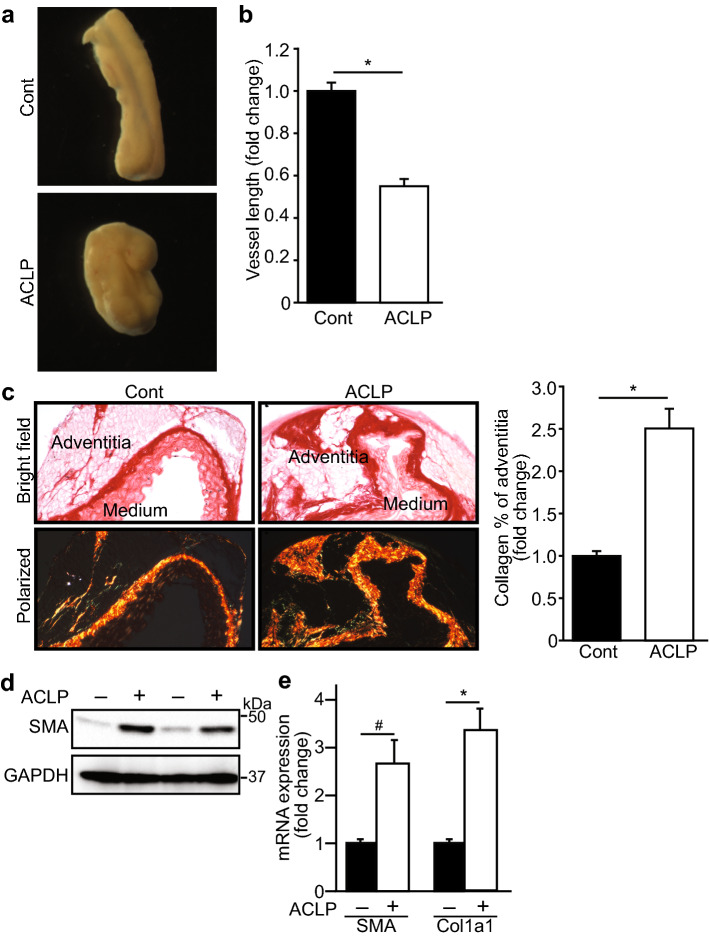
ACLP induces aorta contraction and adventitial fibrosis. Mouse aortas were isolated and cultured in the presence or absence of ACLP 30 nM for 7 days (**a**). After 7 days ACLP treatment, aorta images were taken and quantified by ImageJ. Statistical significance was calculated using the Student’s t test (two tailed) (n = 9) (**b**). Aortas were collected and collagen expression was examined by picrosirius red staining and polarized light (**c**). Quantification of staining results from independent experiments (n = 5) and analyzed by ImageJ. Statistical significance was calculated using the Student’s t test. ^§^*p* < 0.001. RNA and protein were extracted from aortas after ACLP treatment and SMA and Col1a1 expression were examined by Western blot (**d**). RNA were examined by RT-PCR (**e**). Results are presented as the mean ± SEM for 3 independent experiments performed in triplicate. The statistical difference between control and ACLP treatment groups was determined using two-tailed Student’s t test. *p < 0.001, ^#^*p* < 0.005.

To examine ACLP function in an ex vivo model, mouse aortas were isolated and cultured in the presence or absence of ACLP 30 nM for 7 days. We found that aorta lengths were reduced by 45% compared to that in control after ACLP treatment (Fig. 5a,b). Picrosirius staining and quantified using ImageJ revealed that collagen expression was markedly increased in the aortic adventitia treated with ACLP compared to controls (Fig. 5c). Additional sets of aortas were extracted for total protein and we observed an increase in SMA expression (Fig. 5d). We did not detect collagen in these Western blots potentially because it was primarily insoluble. However, analysis of RNA from these cultured vessel fragments resulted in a statistically significant increase in both SMA and Col1a1 mRNA expression (Fig. 5e). These data support the concept that ACLP is a critical mediator of adventitial cell differentiation and remodeling by increasing fibrosis and vessel contraction.

## Discussion

**Figure 6:**
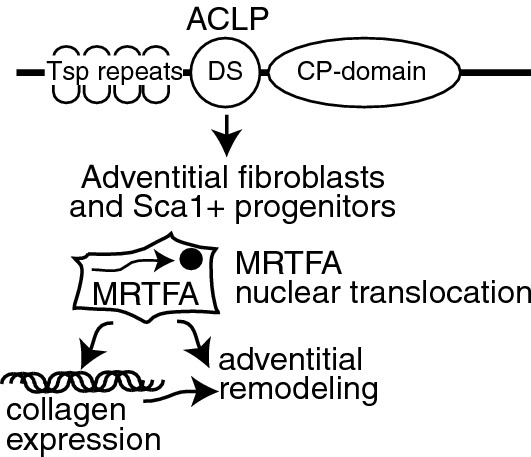
Model of aortic carboxypeptidase-like protein regulation of progenitor and fibroblast differentiation through MRTFA. ACLP structure indicated by predicated thrombospondin (Tsp) repeats, collagen-binding discoidin (DS) domain, and catalytically inactive carboxypeptidase (CP) domain. ACLP stimulates adventitial fibroblasts and Sca1+ progenitors in part through regulating the nuclear translocation of MRTFA. ACLP stimulation results in increases in collagen expression and adventitial remodeling.

In the present study, we demonstrate that ACLP enhances the differentiation of specific adventitial Sca1 progenitors and fibroblasts into myofibroblasts or smooth muscle-like cells. Importantly, ACLP mediates differentiation of adventitial progenitors via stimulating the nuclear translocation of MRTFA and subsequent activity. These studies uncovered a new ACLP regulated pathway that increases adventitial collagen matrix expression and contraction (Fig. 6).

Several screening studies have identified changes in ACLP expression correlating with cardiovascular disease processes. For example, Didangelos et al. found that ACLP degradation is increased in aortic aneurysms compared to controls^30^. ACLP was also identified in the ECM in a porcine model of cardiac ischemia/reperfusion remodeling^29^. In addition, increased ACLP expression parallels with severity of fibrosis in nonalcoholic steatohepatitis and idiopathic pulmonary fibrosis^39,46^. Our previous work determined that ACLP was highly induced in mouse vascular injury^25^ and increasing evidence indicates that progenitor cells exist in adventitia and differentiate into multiple types. ACLP is prominently expressed in the adventitia of mouse femoral arteries following vascular injury (Fig. S1). It has been demonstrated that adventitial Sca1 cells differentiate into collagen-producing cells and contribute to vascular fibrosis and perivascular cardiac fibrosis^20,23^. Several studies indicate that adventital Sca1+ cells are a heterogenous cell populations and contribute to vascular disease progression^24,35,36^. Our initial analysis of ACLP in the vascular adventitia by single cell RNA sequencing revealed that ACLP is co-expressed with Sca1 and also with the fibroblast makers decorin and col1a1 (Fig. S2). Adventitial Sca1 cell differentiation is regulated by ECM, growth factors and a number of cellular receptors^37^. The ECM is an intricate network of proteins that surrounds cells and acts as a key component of stem cell niches and participates in stem cell maintenance, proliferation, self-renewal and differentiation^37,38^. Our work showed that elevated ACLP alters the differentiation of these progenitors by promoting a more smooth muscle and ECM-producing myofibroblast phenotype as observed in vascular disease settings. Our studies are also consistent with a role for ACLP in maintaining vascular homeostatasis as loss of ACLP functions in humans results in a novel variant of Ehlers Danlos Syndrome with aortic dilatation^33^.

The precise signaling mechanisms controlling adventitial progenitor differentiation are not fully elucidated. Earlier studies showed that gene expression changes in all members of the myocardin family with adventitial progenitor differentiation^24^ and myocardin is involved in adventitial Sca1 cell differentiation into smooth muscle cells regulated by c-Myb^47^. It is recognized that ACLP has direct signaling roles via the TGFβ and LRP6/Frz8-β-catenin pathways^48^. There are likely other ACLP-dependent pathways that are integrated with TGFβ signaling and transcriptional networks. Our previous work using inhibition of TGFβ-receptor signaling with kinase inhibitors or siRNA against Smad2/3 repressed ACLP-dependent effects in lung and adipose tissue cells^39,40^. There are complex interelationships with TGFβ signaling, MRTFA, and the Taz/Yap pathways in mesenchymal cells^49^. While previous work determined that the MRTFA signaling pathway is an important mediator of stem cell fate^50,51^, our work determined that the MRTFA pathway contributes to adventitial differentiation. It has been previously shown that MRTFA and its related family members MRTFB and myocardin are regulated in vascular injury and atherosclerosis models^52^. Although we did not examine the regulation or function of these other family members, our loss of function studies with MRTFA (Figs. 2, 4) support the concept that these proteins cannot compensate for the absence of MRTFA. Interestingly, ACLP increased its own expression that was also dependent on MRTFA (Fig. 2b,c) potentially indicating a feed-forward regulatory loop. Important insight into the role of MRTFA and its regulation in different signaling networks has come from studies examining epithelial and endothelial to mesenchymal transformation^53,54^. In addition to integration of TGFβ signaling pathways by MRTFA described above, MRTFA is also regulated by phosphorylation and Rho kinase pathways^50,54,55^. Our work found a connection between ACLP activity and enhanced MRTFA nuclear localization (Fig. 3) and in the absence of MRTFA responses to ACLP were blunted (Fig. 2). It is currently unknown whether ACLP signaling regulates Rho kinase pathways. Taken together these findings add to our understanding of pathways regulating adventitial differentiation.

Treatment of Sca1 progenitors with ACLP downregulated progenitor marker gene expression including KLF4 (Fig. 1d). KLF4 is a zinc finger protein and acts as a transcriptional repressor that blocks SRF/MRTFA-dependent transcription of multiple smooth muscle marker genes^49^. It has been reported that KLF4 is highly expressed in adventitial Sca1 cells and plays a critical role in the maintenance of SMC progenitors^24^. Once Sca1 cells differentiate, KLF4 is down-regulated and cells acquire SMC markers^24,56^. Consistent with previous studies, we found that KLF4 gene expression is diminished after Sca1 cell differentiation. Previous studies have demonstrated regulatory roles for KLF4, MRTFA, and TGFβ signaling in the modulation of vascular smooth musle cells in part through microRNA dependent pathways^57^. While there is a connection between ACLP activity and the downregulation KLF4 expression, the pathways controlling this regulatory step are currently unknown.

In addition to progenitors, fibroblasts and myofibroblasts play important roles in vascular remodeling through their collagen production and contractile abilities that are similar to SMCs^11^. It is widely recognized that vascular smooth muscle cells and myofibroblasts have similar gene expression patterns including the expression of SMA. The vascular SMC and myofibroblasts in their expression of both ECM genes including collagen and contractile genes including SMA and SM22^15^. Irrespective of their designation, the phenotype of the various cell types in the diseased vessel clearly impact vascular disease progression. Our findings support a model where ACLP contributes to adventitial fibroblast and stem cells activation and promotes the transition of fibroblasts to myofibroblasts resulting in vascular ECM contraction and remodeling. Taken together these studies have uncovered a novel pathway where ACLP regulates adventitial progenitor and fibroblast differentiation and ultimately contributes to changes in ECM remodeling. Our results connect ACLP with several critical processes that drive pathological vessel remodeling that may be amendable to inhibition through the development of specific inhibitors.

## Methods

All experiments were performed in accordance with relevant guidelines, regulations, and approval by Boston University School of Medicine’s Institutional Biosafety Committee.

### Mice

ACLP^*flx/flx*^ mice containing loxP sites in the ACLP gene were generated at the University of California Davis mouse biology core and were crossed with the tamoxifen inducible Cre (B6.Cg-Tg) (CAG-cre/Esr1*)5Amc/j, Jackson labs). MRTFA^+/+^ and MRTF^−/−^ mice (generously provided by Dr. Eric Olson)^58^ were crossed with mice harboring the SMA promoter driving mCherry and Col1a1 promoter driving GFP (col3.6GFPtpz), kindly provided by Dr. David Rowe^59^ as described^17^. For the MRTFA studies littermates were used for as controls and 8–12 week old male and female mice were used for Sca1 cell isolation. The Institutional Animal Care and Use Committee at Boston University School of Medicine approved all animal experimental procedures (protocol # 14769).

### Histological analysis

Collagen was examined by picrosirius red staining (Electron Microscopy Sciences) following manufacturer’s instruction. Briefly, aortas were fixed and embedded in paraffin. Tissue sections were stained with picrosirius red for 1 h and sections were mounted for observation under polarized light (Olympus IX70) and results quantitated using ImageJ.

### Aorta contraction assay

Thoracic aortas were isolated from 10 male mice. Each aorta was cut into 2 pieces of equal length, one piece for ACLP treatment, one for control. After culture in the presence or absence of ACLP (30 nM) for 7 days, vessel fragments were imaged using an Olympus, SZX16 microscope and analyzed using ImageJ. The lengths for ACLP treated vessel fragments were normalized to controls.

### Cell culture and treatments

Mouse primary aorta adventitial fibroblasts were isolated from male mouse aorta essentially as described^26^. Briefly, the aortas were dissected and the adventitia was separated from the medial layer, minced, and digested in buffer containing 1 mg/ml collagenase (Worthington) and 0.125 mg/ml elastase (Sigma). Cells were maintained in DMEM containing 10% FBS (Hyclone) and antibiotics. Recombinant ACLP was expressed and purified as described^39^. Recombinant TGFβ was obtained from R&D Systems (240-B-002/CF). Unless otherwise indicated, cells were treated with 30 nM ACLP or 1 nM TGFβ.

### Sca1 cell isolation and culture

Sca1 cells were isolated by anti-Sca1 MicroBead Kit (Miltenyi Biotec) following the manufacturer’s instructions. Briefly, male mice were euthanized and the thoracic aortic adventitia was dissected, minced, and then digested with 1× dispase (BD Falcon), 1 mg/ml type 1 collagenase (Worthington), and 4.5 μg/ml DNase at 37 °C for approximately 30 min. Cell suspensions were filtered through a 40 μm cell strainer (BD Falcon) and centrifuged at 200×*g* for 5 min. Cell pellets were resuspended with MACS buffer and cell numbers were counted, followed by incubation with 10 μl FITC-conjugated monoclonal anti-mouse Sca-1 antibody at 4 °C for 10 min. After washing 3 times, cells were incubated with 20 μl microbeads-conjugated monoclonal anti-FITC antibody for 15 min at 4 °C, and then passed through a magnetic cell sorting (MACS) column to isolate Sca1 cells. The yield of Sca1 cells averaged 5% in these purifications. Purified cells were maintained in DMEM supplemented with 10% FBS.

### Reverse transcription polymerase chain reaction (RT-PCR) assays

Total RNA was extracted by GeneJet RNA purification kit (Thermo) and subjected to reverse transcription using SuperScript II kit (Invitrogen), according to the manufacturer’s instructions. qPCR was performed using Luminaris Color HiGreen High ROX qPCR Master Mix (Thermo), following the manufacturer’s instructions. Quantitative analysis was performed by real-time PCR (ABI 7300). Expression was calculated with the ΔΔC_T_ method using 18S to normalize all samples.

Primers were used as follows (validated primer sequences designed by MGH Primer bank).

**Table:** 

*18S*:	fwd/rev CGGCTACCACATCCAAGGAA	TTTTCGTCACTACCTCCCCG
Sca1 (*Ly6a*):	fwd/rev AGGAGGCAGCAGTTATTGTGG	CGTTGACCTTAGTACCCAGGA
Klf4:	fwd/rev GTGCCCCGACTAACCGTTG	GTCGTTGAACTCCTCGGTCT
CD34:	fwd/rev ATCCCCATCAGTTCCTACCAAT	TGGTGTGGTCTTACTGCTGTC
SMA (*Acta2*):	fwd/rev TGACGCTGAAGTATCCGATAGA	GTACGTCCAGAGGCATAGAGG
SM22 (*Tagln*):	fwd/rev CAACAAGGGTCCATCCTACGG	ATCTGGGCGGCCTACATCA
Col1a1:	fwd/rev CTGGCGGTTCAGGTCCAAT	TTCCAGGCAATCCACGAGC
ACLP/*aebp1*:	fwd/rev TCCCCAACTATGATGACTTGGA	GGGGCTACTACCCTCCTTTTTG

### RNA interference

Synthetic small interference RNA (siRNA) targeting mouse ACLP was purchased from Dharmacon (5′ GGCUCAAGAUCUACGCAAU 3′). A siRNA with a non-targeting sequence (scramble siRNA, Dharmacon) was used as a negative control. The siRNAs were transfected at indicated doses using RNAi Max (Invitrogen) according to manufacturer’s instructions. Primary cells were transfected with siACLP (1, 2 and 4 nM) or siControl (4 nM) and 48 h after transfection, protein was isolated, subjected to Western blot.

### Immunoblot assays

Cells were lysed in extraction buffer (25 mM Tris, pH 7.4, 50 mM sodium chloride, 0.5% sodium deoxycholate, 2% NP-40, 0.2% SDS) containing protease inhibitors. Total cell lysates were separated on 4–20% SDS-PAGE (Invitrogen), transferred to nitrocellulose membranes (Millipore), immunoblotted with antibodies, and visualized using a SuperSignal West Dura Extended Duration Substrate (Thermo Scientific). The antibodies used in this study are anti-SMA (Sigma, A2547, 1:4000 dilution), anti-type I collagen (Rockland, 600-401-103, 1:1000 dilution), anti-MRTFA (Santa Cruz, sc-21558, 1:250 dilution), anti-ACLP (1:4000 dilution)^27^ and anti-GAPDH Sigma, G9545, 1:10,000 dilution). Western blots were quantitated using ImageJ and normalized to GAPDH. The results are expressed as fold change versus control.

### Immunofluorescence staining

Sca1 cells were plated onto glass chamber slides and incubated in the presence or absence of ACLP (30 nM). After 7 days, cells were washed, fixed in 4% paraformaldehyde (PFA), permeabilized with 0.1% Triton X-100, incubated with anti-SMA antibodies (Sigma, A2547, 1:500 dilution) followed by Alexa-Fluor-568 conjugated secondary antibodies (Invitrogen, A11031, 1:500 dilution). Samples were counterstained with DAPI and mounted. Images were taken at identical exposures using an Olympus 1X70 microscope and an Optronics camera and analyzed by ImageJ. To study MRTFA translocation, adventitial fibroblasts were serum starved overnight and treated with ACLP (30 nM) for 2 h and examined by immunofluorescence as described^55,60^. Briefly, after treatment, cells were washed in warm PBS and fixed in 4% paraformaldehyde (PFA). Cells were then incubated with an anti-MRTF-A (1:50; Cat# sc-21558, Santa Cruz Biotechnology Inc., Santa Cruz, CA), followed by the appropriate secondary antibody conjugated to AlexaFluor 568 (1:500; Cat#A-11057, Thermo Scientific, Waltham, MA 02451). Pictures of at least four random fields were captured and analyzed by ImageJ. For each image, distribution of nuclear and cytoplasmic of MRTFA staining was determined and the ratios of the average nuclear and cytoplasmic staining distribution in total cells of each field were calculated using established methods^55^.

### Collagen gel contraction assays

Collagen matrix contraction assay was performed essentially as described^28^. Mouse adventitial fibroblasts isolated from male ACLP^*flx/flx*^ Cre+, MRTFA^+/+^ and MRTF^−/−^ mice. ACLP^*flx/flx*^ Cre+ were treated with tamoxifen in vitro for 3 days to delete ACLP. Tamoxifen was washed out for 2 additional days and fibroblasts were embedded in attached collagen matrices, followed by control, TGFβ, or ACLP treatment overnight. Upon releasing the collagen lattice from the culture dish, the embedded cells become free to contract the deformable collagen lattice and images were taken at the end of incubation. The area of contracted collagen lattices was calculated as a percentage of the surface of the dish.

### Statistical analysis

The results presented are the average of at least three independent experiments. All data are plotted as the mean ± SEM and analyzed using GraphPad Prism software. Statistical significance was calculated using analysis of variance (ANOVA), followed by Tukey’s multiple comparison test or by Student’s t-test as indicated in figure legends. Significance was accepted at p < 0.05.

## Supplementary Information

Supplementary Information.

## Data Availability

Data generated or analyzed during this study are included in this published article and in the Supplementary information.
