# Supplementary material for: Aortic carboxypeptidase-like protein regulates vascular adventitial progenitor and fibroblast differentiation through myocardin related transcription factor A
Source: Sci Rep. 2021 Feb 17;11:3948. doi: 10.1038/s41598-021-82941-7 (PMC7889889; doi:10.1038/s41598-021-82941-7)

Supplemental Information

Aortic carboxypeptidase-like protein regulates vascular adventitial progenitor and fibroblast differentiation through myocardin related transcription factor A

Dahai Wang[1,2], Nabil Rabhi[1], Shaw-Fang Yet[3], Stephen R. Farmer[1], and Matthew D. Layne[1]*

[1]Department of Biochemistry, Boston University School of Medicine, 72 E. Concord St, Boston, MA, 02118 USA

[2]Current address: Department of Hematology, Boston Children's Hospital, Boston, MA, USA

[3]Institute of Cellular and System Medicine, National Health Research Institutes, Zhunan 35053, Taiwan

*Corresponding author

mlayne@bu.edu; Tel # 617-358-4409

Supplemental Methods:

Mouse femoral artery injury model and immunohistochemistry

Femoral artery injury experiments and analysis was performed as described[1] using both male and female mice in accordance with the guidelines and regulations of the Institutional Animal Care and Use Committee at Harvard Medical School and Boston University School of Medicine. Immunohistochemistry of vessel sections with smooth muscle alpha actin and ACLP were performed as described[1,2].

Characterization of adventitial cells by single cell RNA sequencing

Thoracic adventitia was collected from male mice and processed using a tissue dissociation kit (Miltenyi Biotec). Cells were prepared for single-cell sequencing according to the 10x Genomics protocols in the Boston University Medical Center Single Sequencing Core. Sequencing was performed on Illumina NextSeq500. The Cell Ranger Single-Cell Software Suite (v.3.1.0) was used to perform sample demultiplexing, barcode processing, single-cell 3′ counting, and counts alignment to mm10 mouse reference genome. The filtered matrices produced by Cell Ranger pipeline were loaded into the Loupe Cell Browser for clustering and to examine genes of interest.

References

1. Wei, J. *et al.* Increased neointima formation in cysteine-rich protein 2-deficient mice in response to vascular injury. *Circ Res* 97, 1323-1331, doi:10.1161/01.RES.0000194331.76925.5c (2005).

2. Layne, M. D. *et al.* Characterization of the mouse aortic carboxypeptidase-like protein promoter reveals activity in differentiated and dedifferentiated vascular smooth muscle cells. *Circ Res* 90, 728-736, doi:10.1161/01.res.0000013289.97650.c8 (2002).

Supplemental Figure 1

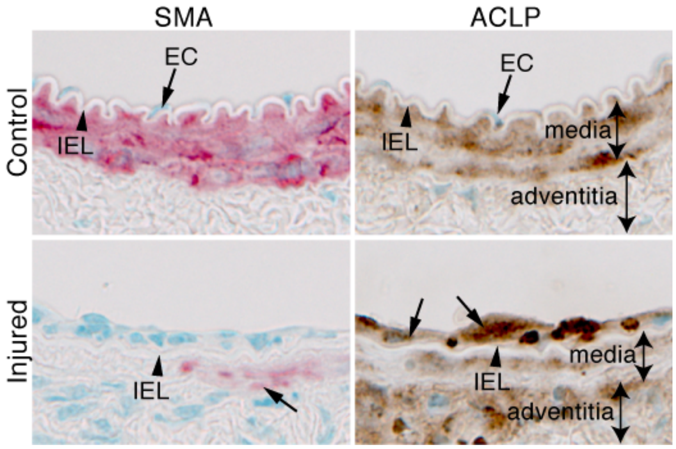

Figure S1. ACLP expression in the vascular adventitia following femoral artery injury. The femoral arteries of male mice were injured and harvested after 7 days and stained. Control and injured histological sections with stained with SMA or ACLP. Expression of ACLP in the early neointima of injured femoral arteries. Note expression in adventitial layer following injury. SM α-actin (SMA) staining (red) in control (uninjured) vessel identifies differentiated SMC (lumen is at top). EC endothelial cell, IEL: internal elastic lamina.

Supplemental Figure 2

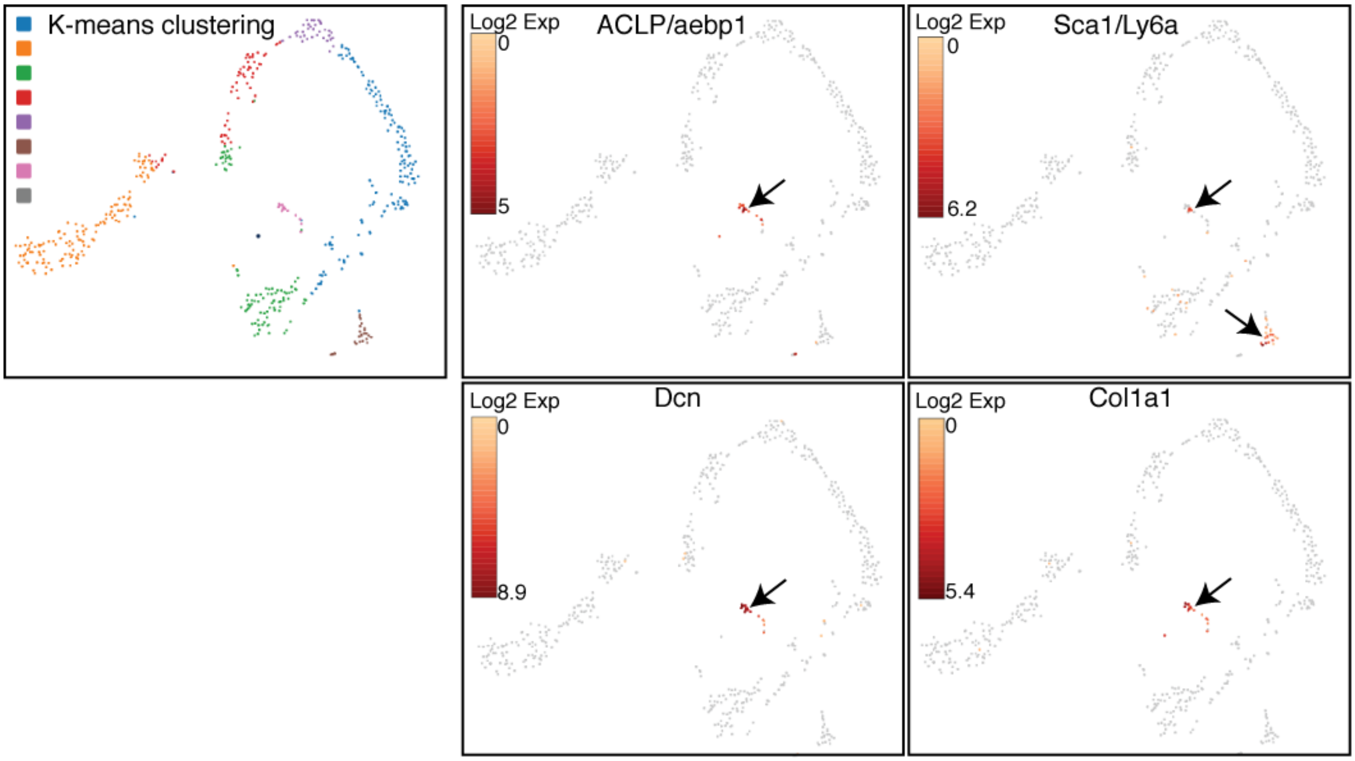

Figure S2. Expression of ACLP in adventitial Sca1+ cells and fibroblasts. Thoracic aorta adventitial cells were collected from male mice were prepared for single-cell sequencing. Expression of ACLP/aebp1, Sca1/Ly6a, and the fibroblast markers decorin (Dcn), and collagen 1 (Col1a1) were assessed.

Figure S3 Full-length Western blots.

Fig. 1f— Western blots

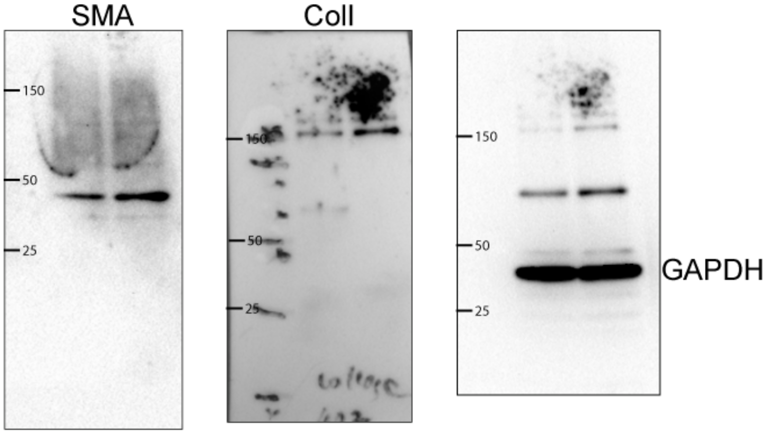

Fig 2b—Western blots

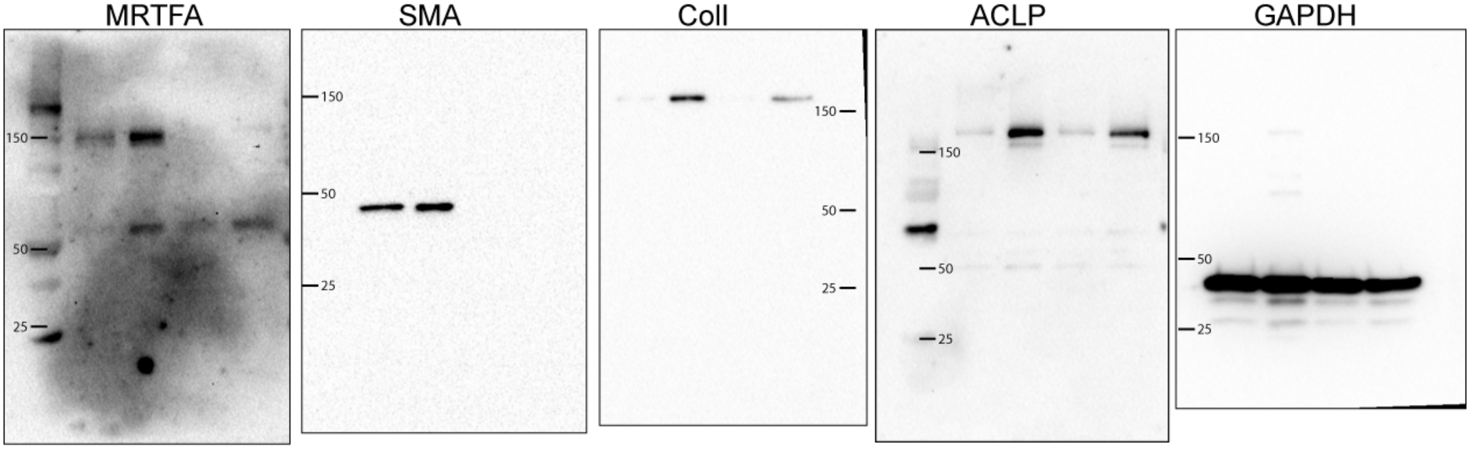

Fig 3a—Westerns blot

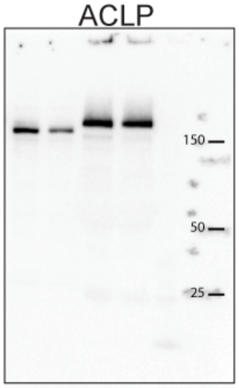

Fig 3c—Western blots

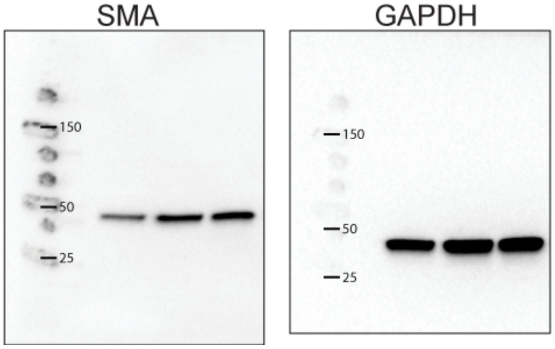

Fig 3d—Western blots

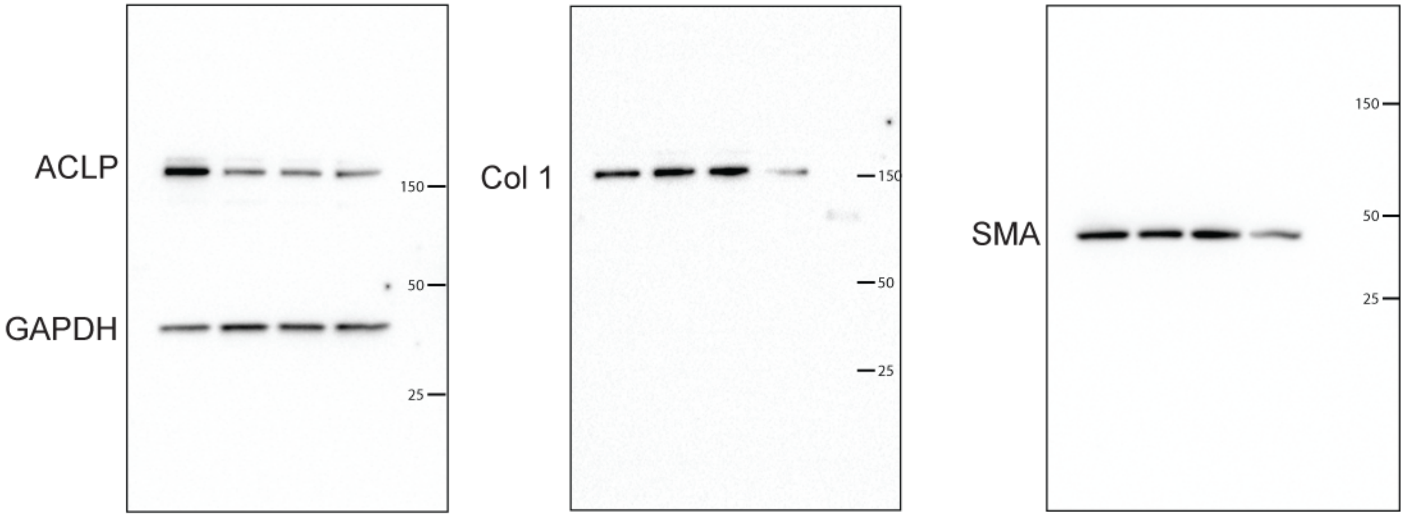

Fig 4e— Western blots

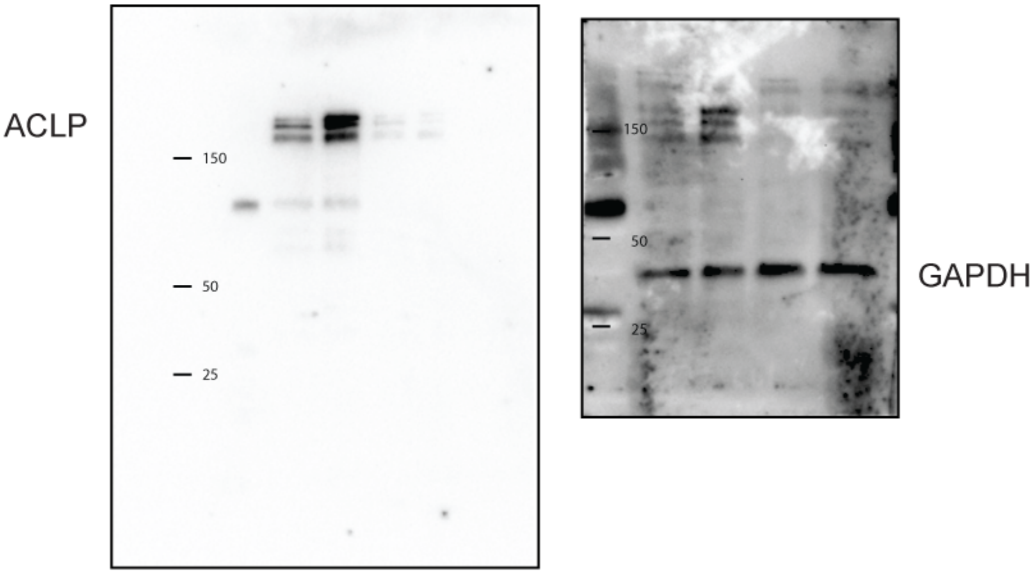

Fig 5d— Western blots

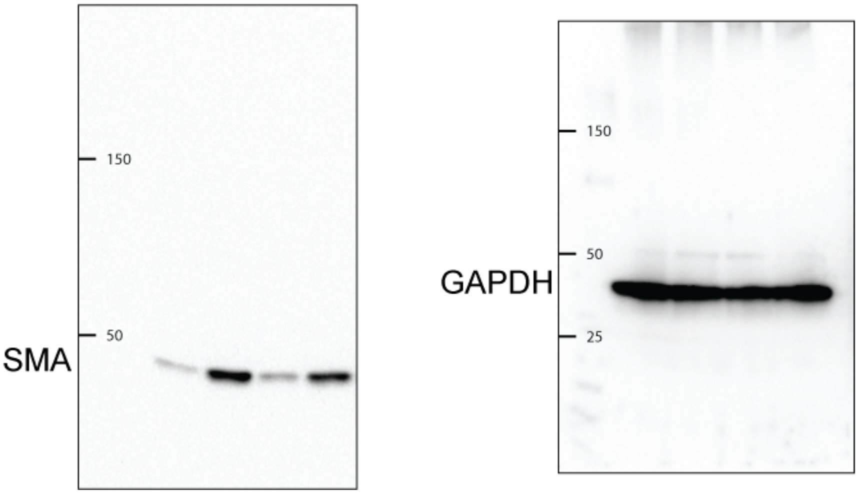

Supplement: Supplementary file 1 — Supplementary Information. [file 41598_2021_82941_MOESM1_ESM.pdf]
